# Endocardial biventricular defibrillator implantation in a patient with superior vena cava obstruction

**DOI:** 10.1093/europace/euv052

**Published:** 2016-02-25

**Authors:** Malcolm C. Finlay, Kulvinder Lall, Ross J. Hunter, Richard J. Schilling

**Affiliations:** St Bartholomew's Hospital, West Smithfield, London EC1 7BE, UK

A 41-year-old female with previous myocardial infarction and subsequent late cardiac arrest presented with serial biventricular defibrillator generator site infections. Following system extractions, intrinsic QRS duration was 140 ms, LV ejection fraction 14%, and NYHA class was III. Superior vena cava obstruction was confirmed with venography.

A novel direct transatrial endocardial approach for reimplant was undertaken, guided by video thoracoscopy, transoesophageal echo, and fluoroscopy, with right-sided thoracic working ports.

Right atrial access was obtained using an Endry's coaxial needle (Cook Cardiology) through an 8 French introducer. This was exchanged for an 8.5 French steerable sheath; an active fixation defibrillator lead was placed on the RV septum. Atrial access was repeated, atrial transseptal puncture performed and used to deliver an active lead to the lateral LV endocardium via a steerable sheath (Thoracoscopic view, *Panel A*). A third atrial puncture delivered an endocardial right atrial lead. Leads were tunnelled subcutaneously to a left prepectoral generator (Protecta XT, Medtronic, final lead positions shown in CT reconstruction, *Panel B*).

Postoperative paroxysms of atrial fibrillation responded to amiodarone and bilateral pericardial effusions were drained. She was discharged 2 weeks following implant. Eleven months hence, the patient has had no further hospital admissions or arrhythmic events.

The full-length version of this report can be viewed at: http://www.escardio.org/Guidelines-&-Education/E-learning/Clinical-cases/Electrophysiology/EP-Case-Reports.


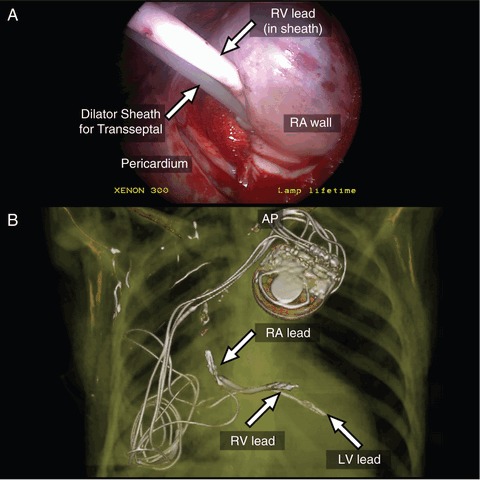


## Funding

Funding to pay the Open Access publication charges for this article was provided by Queen Mary University of London and NIHR.

